# Intranasal insulin for improving cognitive function in multiple sclerosis

**DOI:** 10.1016/j.neurot.2025.e00581

**Published:** 2025-04-18

**Authors:** Scott D. Newsome, Kathryn C. Fitzgerald, Abbey Hughes, Meghan Beier, Jacqueline Koshorek, Yujie Wang, Daniela Pimentel Maldonado, Thomas Shoemaker, Taimur Malik, Tarik Bayu, Pablo E. Ravenna, Ama Avornu, Elias S. Sotirchos, Meghan Romba, John Muschelli, Todd T. Brown, Peter A. Calabresi, Ellen M. Mowry

**Affiliations:** a Johns Hopkins University School of Medicine, Department of Neurology, Baltimore, MD, USA; b Johns Hopkins University School of Medicine, Division of Rehabilitation Psychology and Neuropsychology, Baltimore, MD, USA; c Johns Hopkins Bloomberg School of Public Health, Department of Biostatistics, Baltimore, MD, USA; d Johns Hopkins University School of Medicine, Division of Endocrinology, Baltimore, MD, USA

**Keywords:** Multiple sclerosis, Cognitive dysfunction, Insulin, Clinical trials, Intranasal treatments

## Abstract

Cognitive impairment is common in people with multiple sclerosis (PwMS). There is an urgent need to identify/develop novel therapies that can help cognitive function in MS. Insulin is critical for helping with regulation of multiple CNS functions, including learning and memory. Insulin administrated intranasally has shown to improve memory and learning in healthy people and in those with some neurodegenerative disorders. Hence, there was rationale for investigating intranasal insulin in PwMS who experience cognitive impairment. We completed a phase Ib/II, randomized, double-blind, placebo-controlled trial; participants were randomized in a 1:1:1 fashion, stratified by relapsing versus progressive MS, to intranasal insulin 10 ​international units (IU) twice a day, 20 IU twice a day, or placebo for 24 weeks. One-hundred and five PwMS were enrolled, 69 of whom had at least one follow up visit during the active treatment phase of the trial (baseline to week 24). The cohort's mean age was 52.4 ​± ​9.7years, 62 ​% were female, and ∼60 ​% had relapsing-remitting MS. The most common side effects amongst treatment groups included headache, rhinorrhea, and dizziness. There were 13 SAEs which were not deemed study drug related; there were no deaths. The main clinical outcome measure, SDMT, did not demonstrate any difference between intranasal insulin and placebo. Similar findings were noted for all secondary outcome measures. Intranasal insulin appeared safe and well-tolerated in PwMS. However, it was not superior to placebo in any of the clinical outcome measures assessed, which could have been impacted by the duration of the trial, small sample size for a three-arm trial design, data missingness (particularly during COVID-19), outcome measure insensitivity to change, baseline cognitive reserve, or other factors. Nonetheless, intranasally-administered therapeutics may be of interest to develop further as a way to get across the blood brain barrier.

## Introduction

Multiple sclerosis (MS) is a common and chronic disease of the central nervous system (CNS) featuring inflammation, demyelination, and neurodegeneration. Impaired cognitive function is present in over 60 ​% of individuals with MS and is associated with reduced quality of life, employment loss, social relationship disruptions, driving safety concerns, and issues with therapeutic adherence [[1], [2], [3], [4], [5], [6], [7], [8], [9]]. Impaired cognitive functioning may occur early in the disease and can worsen longitudinally [[10], [11], [12], [13]].

To date, pharmacologic interventions for cognitive impairment in people with MS (PwMS) have had disappointing results [[14], [15], [16], [17], [18]]. While behavioral interventions show promise, they are understudied and are often time consuming, costly, and not widely available [19]. Hence, there is an urgent need to identify or develop novel therapies that improve cognitive function in MS.

Insulin is critical for helping with regulation of multiple CNS functions including brain metabolism, neurite outgrowth, neurotransmitter channel activity, neuronal survival, and learning and memory [20,21]. There are insulin receptors throughout the brain, with robust concentrations located in eloquent areas including the olfactory bulb, hippocampus, cerebral cortex, and cerebellum. Insulin is present at high levels in the brain, and when these levels are decreased, there may be learning and memory impairments [20,22,23]. The cognitive impairment that can ensue in the context of low brain insulin levels may be related to its ability to protect neurons from various insults including oxidative stress, ischemia, and glutamate-related excitotoxicity [20]. Moreover, insulin's anti-inflammatory effects may also impact brain health via suppressing concentrations of chemokines, cytokines, and other molecules that may provoke ongoing CNS inflammation and damage in disease states [20].

Intranasal insulin has been safe and well-tolerated in many human trials. Intranasal insulin was shown to have neuroprotective and restorative effects in several human clinical trials [[24], [25], [26], [27], [28], [29], [30], [31], [32], [33], [34], [35], [36], [37], [38], [39], [40]]. Insulin signaling in neurons exerts a neuroprotective effect, which has been seen in models of Alzheimer's, Parkinson's, and Huntington's Diseases [41]. In a randomized, double-blind, placebo-controlled study of people suffering from amnestic mild cognitive impairment or Alzheimer's Disease (AD), intranasal insulin preserved cognitive function compared to placebo [24]. The insulin-treated patients also did not show a decline in measures of brain metabolism, whereas the placebo-treated participants did show decreases in fluorodeoxyglucose ^18^F uptake in various lobes (parietotemporal, frontal, precuneus, and cuneus). Further, improvements in episodic memory persisted for several weeks after treatment cessation. Given these findings, we proposed and conducted a randomized controlled trial of intranasal insulin in cognitively-impaired PwMS.

## Materials and Methods

### Trial design

**Fig. 1:**
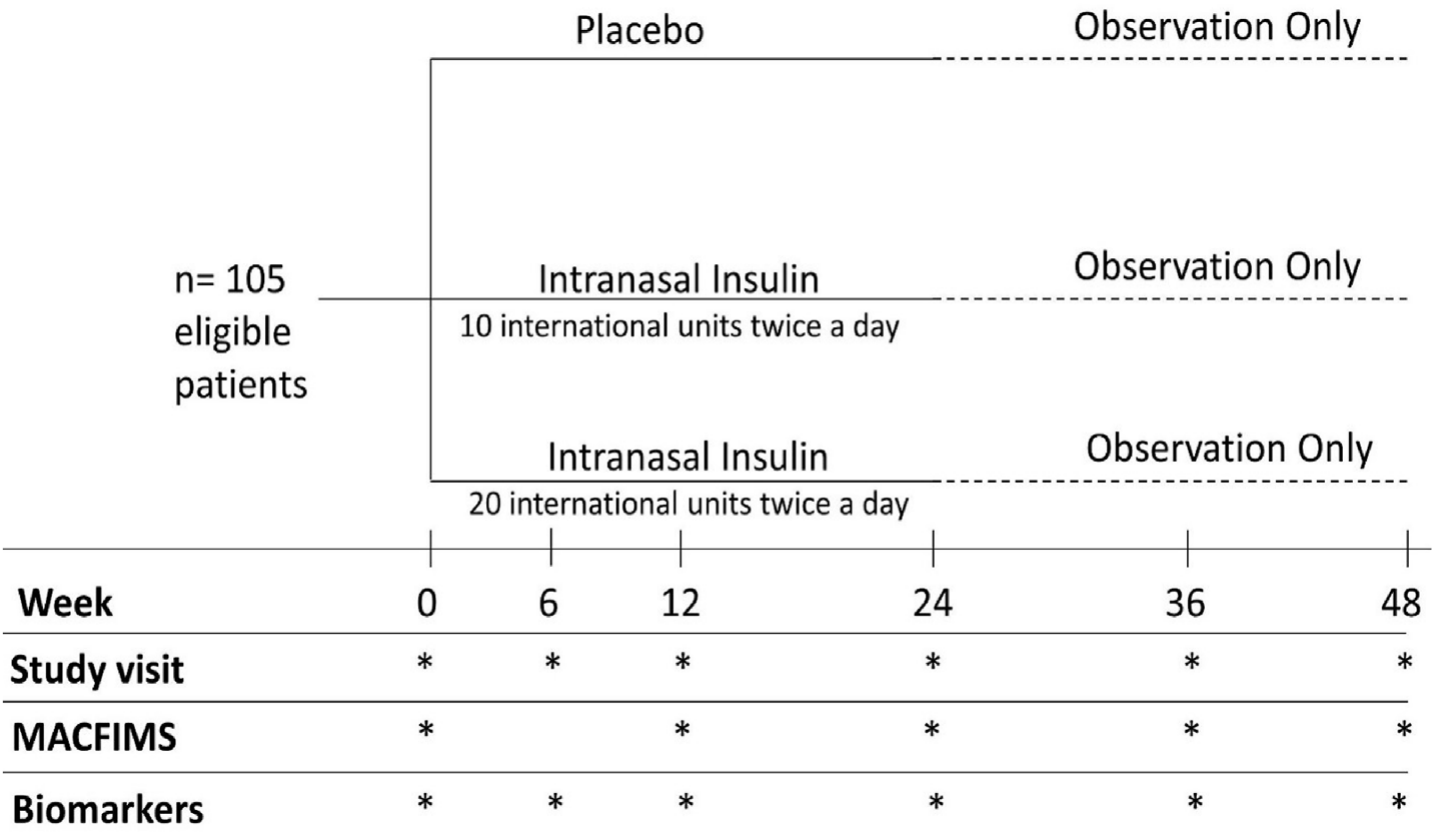
Original trial design.

This was a randomized, double-blind, placebo-controlled phase Ib/II study (NCT02988401) that was performed at a single center- Johns Hopkins MS Precision Medicine Center of Excellence. Participants were randomized to intranasal insulin 10 ​international units (IU) twice a day, 20 IU twice a day, or placebo twice a day for 24 weeks in a 1:1:1 fashion (Fig. 1). Insulin was administered intranasally using the novel ViaNase™ controlled particle dispersion system, allowing for direct delivery of the medication to the nasal epithelium, leading to maximal transport to the CNS (Kurve Technology, Lynnwood, WA) [24].

Institutional review board (IRB) approval was secured before enrollment began, and all participants provided written informed consent before enrolling. An independent data and safety monitoring board (DSMB), consisting of an MS specialist (MR), endocrinologist (TTB), and statistician (JM), met twice yearly to review enrollment progress, protocol deviations and safety data. A DSMB meeting was also convened any time a serious adverse event (SAE) was reported. A medical monitor was appointed to provide independent safety review of unanticipated problems and SAEs.

The trial began enrolling in January 2018, and the last person was enrolled in January 2021. Due to slow recruitment, the eligibility criteria were modified during the early part of the study with expert guidance from our trial's neuropsychologists (AH and MB). The main modifications included; 1) participants needing at least 1.0 standard deviation or greater below published mean Symbol Digit Modalities Test (SDMT) z-score at screening and baseline visit (versus 2.0), 2) main initial medication exclusions included a large list of medications that may alter cognitive performance and were modified to allow for a broader group of participants to enroll, as long as medications were stable in dose and frequency leading up to screening (see Table 1).

**Table 1:** Eligibility criteria and rationale for the trial.

Eligibility Criteria	Rationale
Meets 2010 criteria for multiple sclerosis (MS)	Study is focused on individuals with MS
No relapse in past 3 months	To avoid relapse-induced cognition confounding
Age 18–70 years	To ensure results generalizable
At least mild cognitive impairment (1.0 standard deviation or greater below the published mean SDMT z-score, or a score of <34 on the processing speed test [PST])	To ensure that we are able to detect improvements in cognition if insulin does in fact have this effect
Capacity to learn and self-administer intranasal insulin, or presence of a caregiver with such capacity who is willing to do it for the duration of the trial	To ensure that subjects are able to take the medication as prescribed
Untreated or on the same MS disease-modifying therapy for at least 2 months, with no anticipated change in the next year	To avoid change in measures due to change in medication
No current, active major depression	May impact results of cognitive testing
No tricyclic antidepressant or anticonvulsant (except carbamazepine, pregabalin or gabapentin) use within 6 weeks of screening; if on oxybutynin or tolterodine, on stable dose for >6 months without plans for changing dose in the next year	May impact results of cognitive testing
If taking selective serotonin (±norepinephrine) reuptake inhibitors, pregabalin, gabapentin, sympathomimetic, monoamine oxidase inhibitor, antipsychotic, amantadine, cholinesterase inhibitor, memantine, modafinil, armodafinil, or evening short-acting benzodiazepines, on stable dose for 6 weeks or greater	To minimize impact on cognitive testing without impairing recruitment/limiting generalizability (MS patients use these therapies commonly)
Not pregnant or nursing, and willing to prevent pregnancy during study if of childbearing potential	Risks of intervention to developing fetus or breastfeeding infant unknown
No tetrahydrocannabinol use in past 6 weeks; no other illicit drug or alcohol abuse in past 3 months	To ensure participants can adhere to treatment/avoid impact of substances on cognitive testing
No known history of diabetes mellitus or insulin resistance	To avoid confounding due to global glucose dysregulation or use of systemic insulin
No active liver disease, stage IV/V kidney disease or severe metabolic derangements	To prevent differential metabolism of insulin
No central nervous system disorder other than MS or headache	To avoid confounding due to insulin effect on other disorder
No issue making participation not in best interest of patient	To prevent any undue harm to patient

The trial's conduct was substantially disrupted by the coronavirus disease 2019 (COVID-19) pandemic; all human research activities were halted during the early part of the pandemic, including study enrollment, consistent with local regulations and with the recommendation of the DSMB. The trial protocol was modified, per the institutional IRB, to minimize the degree of in-person interactions needed to support the primary objectives of the trial. These changes are detailed in the Supplementary Materials. When the COVID-19 pandemic began, 13 study participants were in the active treatment phase (first 24 weeks) of the study. These individuals were asked to stop the study drug given the lack of data surrounding the safety of inhaled products with respect to risk of contracting COVID-19, per the determination of the DSMB. However, as no such risk emerged over time, our DSMB felt that it was safe to resume enrollment once approved by the IRB. Enrollment resumed in July 2020 and was completed in January 2021.

### Study population

A total of 105 individuals, aged 18–70 years, who met 2010 McDonald criteria for MS and demonstrated at least mild cognitive impairment (1.0 standard deviation or greater below the published mean SDMT z-score, or a score of <34 on the processing speed test [PST]) were included in the trial. Participants had no MS relapse in the 3 months prior to enrollment, had not had a change in their MS disease-modifying therapy (or lack thereof) within the previous 2 months, and were not anticipated to have a change thereof in the subsequent year. Those with current, active major depression (assed via Beck Depression Inventory and self-report) or with self-reported current diabetes or insulin resistance were excluded. A variety of psychoactive medications with recent (and, for some, anticipated) dosage changes were exclusionary, although participants with recent changes could be re-screened after those criteria were met. Complete eligibility criteria are outlined in Table 1.

### Randomization and masking

Intranasal insulin (Novolin R, Novo Nordisk, Princeton, NJ) was evaluated in two doses (10 IU twice a day, 20 IU twice a day; dosages consistent with a prior trial in AD)^24^ and compared to the placebo with sterile diluent (Eli Lilly and Company – NDC:00002–0800) for masking. The 1:1:1 randomization was created by the study biostatistician (KCF); randomization was stratified by relapsing versus progressive MS, as adjudicated from the participant's clinical chart by the study coordinator and/or a treating physician at screening/baseline visit.

The randomization schedule was provided directly to the Johns Hopkins Investigational Drug Service by the study biostatistician to ensure masking of the remaining trial personnel. As noted above, the sterile diluent was used to preserve the blinding, since it has an odor similar to that of insulin (in diluent). The medications were prepared by the Johns Hopkins Investigational Drug Service and appeared identical to maintain masking.

### Administration of study drug

Consistent with a prior trial in AD, a nasal drug delivery device (Kurve Technology, Lynnwood, WA) was utilized to ensure optimal exposure of the olfactory epithelium [24].

The pertinent study team members underwent training on how to use the nasal device and perform the study drug administrations along with troubleshooting and identifying more common drug delivery device malfunctions. Step-by-step instructions were provided to study subjects, including a picture reference guide for visual instructions. In order to ensure subjects (or their responsible caregivers) were able to use the medication as instructed, all subjects and caregivers (when relevant) were instructed on the use of the inhaler and observed by the study coordinator and/or treating physician when performing their first dose administration at the time of the baseline visit. Supplementary teaching was provided to individual subjects as needed.

Subjects were instructed to take the study drug intranasally twice a day (approximately 12-h in-between each dose) using the step-by-step instructions. If a dose was missed, subjects were instructed to take it as soon as they could if it was within a few hours of the missed dose; otherwise, they resumed with the next scheduled dose. Subjects were instructed to record missed doses and time of administration within their study diaries.

### Study outcomes

#### Safety and tolerability

From a safety perspective, we monitored the first 15 participants for a period of 90 ​min after their first dose of the study medication to evaluate finger stick blood glucose levels to evaluate for meaningful change in circulating glucose levels.

We recorded treatment-emergent AEs reported by participants, as collected through a provided diary and/or as directly reported to study team members. The study team was in frequent communication with study participants to assure complete safety data were captured. We used the National Cancer Institute's Common Terminology Criteria for AEs version 4.0 to report and grade all adverse events, whether or not they were related to treatment. Investigators recorded all study AEs in the chart, observing them until they resolved or stabilized. AEs were collected from the start of the study until a participant completed the study. The AEs that were unresolved at the time of study termination were followed until they resolved or up to 30 days. The study medication was discontinued if an AE grade 3 or higher occurred and at least possibly related to the medication or if the subject could not tolerate the medication/wanted to discontinue it.

SAEs were captured as directly reported to study personnel during frequent check-ins with study team members. Additionally, hospital admissions within the Johns Hopkins Health System were captured via automated notifications within the electronic health record to one of the study physicians. SAEs were collected from informed consent signing until 30 days after study completion or until 30 days after a participant withdrew from the study. Reporting SAEs followed the International Conference on Harmonisation guidelines and the IRB and DSMB were notified within two business days of reporting/discovering the SAE. In addition, the Columbia Suicide Severity Rating Scale was administered at each study visit.

### Clinical outcomes

We used the Minimal Assessment of Cognitive Function in MS (MACFIMS) battery which includes: oral SDMT**,** commonly used in MS to assess processing speed (with a 3.5 to 4 point raw score difference considered as clinically meaningful) [1,6,[42], [43], [44], [45]]; the Controlled Oral Word Association Test (COWAT) as a measure for phonemic fluency [46]; the California Verbal Learning Test, Second Edition (CVLT-II**)** as a verbal learning and memory test [47,48]; the Brief Visuospatial Memory Test-Revised (BVMT-R**)** as a visual, nonverbal test of learning and memory [49]; the Rao-version of the Paced Auditory Serial Addition Test (PASAT) to evaluate processing speed, working memory, and basic addition skills [46]; the Judgment of Line Orientation Test (JLO) to assess visual-spatial abilities [46,50]; and the Delis-Kaplan Executive Function System (DKEFS) to test executive functioning, concept formation, and cognitive flexibility [51]. The main clinical outcome measure for this study was SDMT. For participants missing baseline SDMT, screening SDMT was used.

Additional clinical outcome measures included the other components of the MACFIMS, the Multiple Sclerosis Functional Composite (MSFC) score, health-related quality of life (Functional Assessment of Multiple Sclerosis [FAMS]), and the Expanded Disability Status Scale (EDSS) [[52], [53], [54], [55]]. We also evaluated how overall sleep quality in people with MS impacts health-related quality of life with the Pittsburgh Sleep Quality Index (PSQI) [56].

### Statistical analyses

Safety data were captured in real time and assessments focused on AEs with incidence rate of AEs recorded by system organ class, severity, and by relationship to the study treatment.

For quantitative parameters, summary statistics for measures and change over the course of the study are presented in table format. We calculated descriptive statistics overall and across treatment group based on the variable in question (e.g., means and standard deviations, median and interquartile range, and percentages).

For analytic models for each cognitive outcome, the primary hypothesis test compared insulin treatment (regardless of dose) versus placebo and was conducted first. Secondary pairwise tests comparing insulin dose (high versus low) versus placebo and high versus low insulin dosages on cognitive outcomes were subsequently performed. For our primary analysis, we assessed change in SDMT over time using a linear mixed effects model with unstructured covariance structure, and random intercepts. Similar models were fit for other cognitive tests (and MS functional scores like component tests for the MSFC, health-related quality of life, depression and sleep-based outcomes). All analyses were adjusted for age, sex, and years of education.

Stratified analyses were also performed due to the COVID-19 pandemic for the outcomes of interest. The pre-COVID-19 time period was considered as all study visits occurring before March 2020 and the COVID-19 shutdown and beyond was considered from after March 2020. Additional pre-specified stratified analyses were performed that including other key covariates of interest; disability status, history of depression, number of non-MS medications (<5, ≥5), MS subtype (relapsing-remitting MS [RRMS], progressive MS), and whether on a DMT or not.

### Role of the funding source

There was one funding source (Department of Defense; W81XWH-16-1-0693) for this clinical trial. The design, collection and data analyses, along with interpretation of the data and writing of the manuscript were not performed or influenced by the funder. In addition, all authors had full control over data and accept full responsibility of this manuscript.

## Results

### Demographics and clinical characteristics

**Fig. 2:**
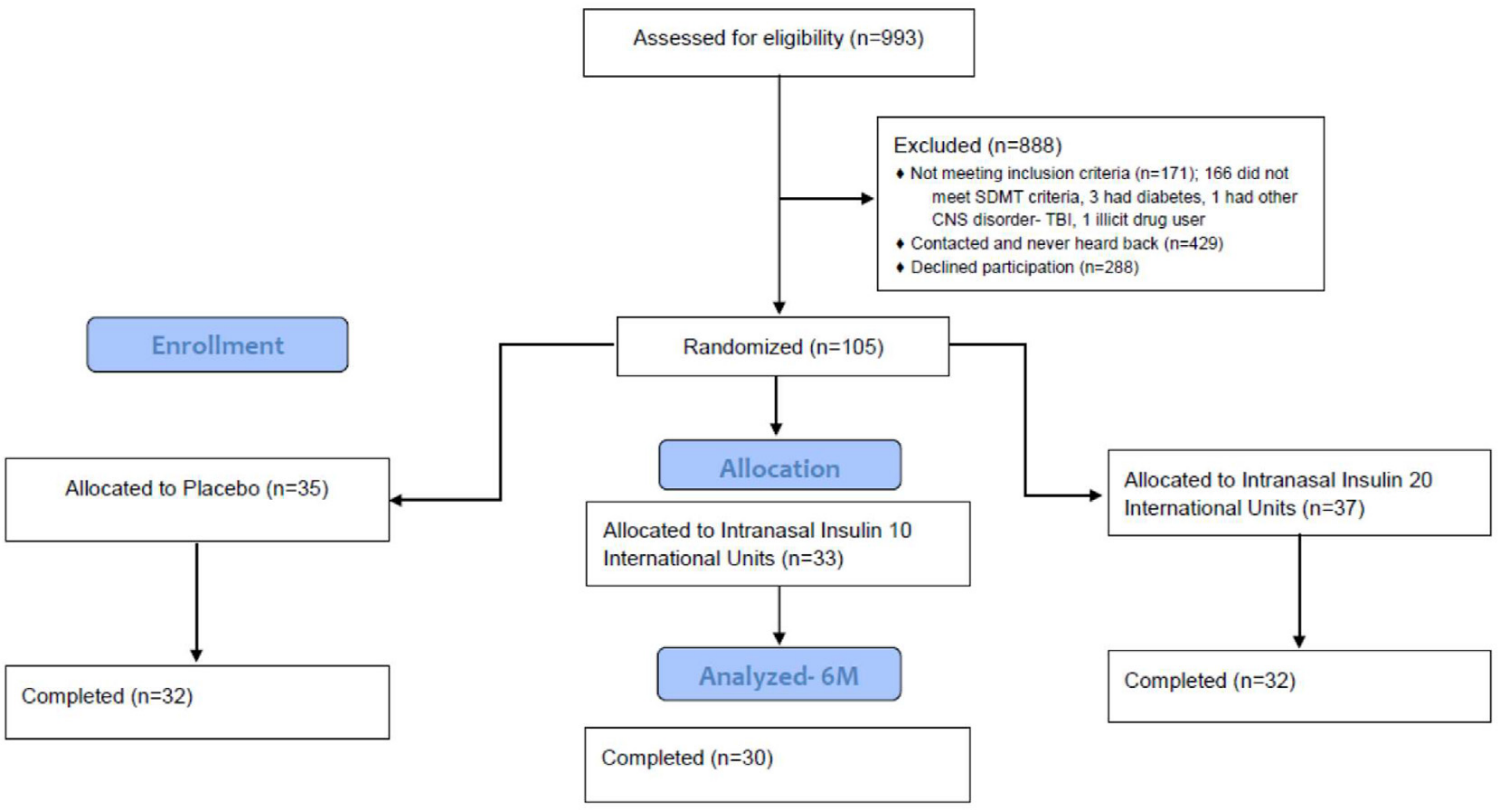
CONSORT diagram.

We screened 559 potentially eligible patients; 288 declined participation; another 166 did not meet cognition score eligibility criteria (Fig. 2-CONSORT diagram). A total of 105 eligible patients with MS enrolled in the study, 69 of whom had at least one follow up visit during the active treatment phase of the trial (baseline visit to week 24 visit).

Full baseline demographics and clinical characteristics of the study population per treatment group are represented in Table 2. The entire cohort had a mean age of 52.4 ​± ​9.7 years, 62 ​% were female, ∼60 ​% had RRMS and screening median (interquartile range) EDSS was 4.0 (3.0–6.0). There were no baseline differences between treatment groups including age, sex, race, disability status, or body mass index.

**Table 2:** Descriptive characteristics of study participants by INI dose.

Characteristic	Overall, N ​= ​105^a^	Placebo, N ​= ​35^a^	LDINI, N ​= ​33^a^	HDINI, N ​= ​37^a^
**Randomization arm**				
Placebo	35 (33 ​%)	35 (100 ​%)	0 (0 ​%)	0 (0 ​%)
INI	70 (67 ​%)	0 (0 ​%)	33 (100 ​%)	37 (100 ​%)
**Age, years**	52.4 (9.7)	49.4 (9.2)	54.4 (9.2)	53.5 (10.1)
**Sex, n (%)**				
Female	65 (62 ​%)	22 (63 ​%)	23 (70 ​%)	20 (54 ​%)
Male	40 (38 ​%)	13 (37 ​%)	10 (30 ​%)	17 (46 ​%)
**Race, n (%)**				
Black	31 (30 ​%)	11 (31 ​%)	8 (24 ​%)	12 (32 ​%)
White	74 (70 ​%)	24 (69 ​%)	25 (76 ​%)	25 (68 ​%)
**Latinx, n (%)**				
Latinx	2 (1.9 ​%)	1 (2.9 ​%)	0 (0 ​%)	1 (2.7 ​%)
Non-latinx	101 (96 ​%)	33 (94 ​%)	33 (100 ​%)	35 (95 ​%)
Unknown	2 (1.9 ​%)	1 (2.9 ​%)	0 (0 ​%)	1 (2.7 ​%)
**Weight, pounds**	181.9 (45.3)	182.7 (31.7)	176.2 (49.9)	186.3 (52.2)
**Body Mass index, kg/mˆ2**	28.9 (6.7)	29.1 (4.8)	28.4 (7.2)	29.1 (7.8)
Unknown	1	0	0	1
**Insulin level, mCu/dL**	12.2 (13.6)	12.3 (11.7)	12.0 (15.8)	12.1 (13.7)
Unknown	3	0	3	0
**Fasting glucose, mg/dL**	89.5 (11.8)	89.3 (8.6)	88.2 (12.4)	90.7 (14.0)
Unknown	1	0	0	1
**Highest level of education achieved, n (%)**				
< ​High school	6 (5.7 ​%)	2 (5.7 ​%)	3 (9.1 ​%)	1 (2.7 ​%)
high School	10 (9.5 ​%)	4 (11 ​%)	3 (9.1 ​%)	3 (8.1 ​%)
some College	27 (26 ​%)	11 (31 ​%)	8 (24 ​%)	8 (22 ​%)
college/Graduate	62 (59 ​%)	18 (51 ​%)	19 (58 ​%)	25 (68 ​%)
Unknown	0 (0 ​%)	0 (0 ​%)	0 (0 ​%)	0 (0 ​%)
**Number of comorbidities, n**	4.6 (3.2)	4.3 (3.3)	5.2 (3.1)	4.2 (3.2)
**Number of concomitant medications**	4.1 (3.5)	4.5 (3.6)	4.2 (3.4)	3.7 (3.4)
**BDI-II score**	11.9 (8.2)	12.1 (6.8)	12.0 (7.8)	11.7 (9.8)
Unknown	22	8	8	6
**Subtype, n (%)**				
PMS	10 (10 ​%)	5 (15 ​%)	3 (10 ​%)	2 (5.7 ​%)
RRMS	89 (90 ​%)	29 (85 ​%)	27 (90 ​%)	33 (94 ​%)
Unknown	6	1	3	2
**Disease duration, years**	17.7 (10.9)	16.0 (7.5)	19.7 (12.1)	17.4 (12.4)
**Cognitive symptom duration, years**	7.9 (8.3)	8.0 (6.9)	8.5 (8.8)	7.2 (9.2)
Unknown	1	0	0	1
**Using DMT, n (%)**	85 (82 ​%)	28 (82 ​%)	25 (76 ​%)	32 (86 ​%)
Unknown	1	1	0	0
**DMT class, n (%)**				
Infusion	45 (50 ​%)	15 (48 ​%)	12 (44 ​%)	18 (56 ​%)
Injectable	15 (17 ​%)	7 (23 ​%)	3 (11 ​%)	5 (16 ​%)
None	2 (2.2 ​%)	1 (3.2 ​%)	1 (3.7 ​%)	0 (0 ​%)
Oral	27 (30 ​%)	8 (26 ​%)	10 (37 ​%)	9 (28 ​%)
Other	1 (1.1 ​%)	0 (0 ​%)	1 (3.7 ​%)	0 (0 ​%)
Unknown	15	4	6	5
**EDSS**	4.6 (1.7)	4.2 (1.8)	4.7 (1.8)	4.8 (1.4)
Unknown	1	0	0	1

LDINI ​= ​low dose intranasal insulin; HDINI ​= ​high dose intranasal insulin; BDI-II= Beck Depression Inventory-II; PMS ​= ​progressive multiple sclerosis; RRMS ​= ​relapsing remitting multiple sclerosis; DMT ​= ​disease-modifying therapy; EDSS ​= ​Expanded Disability Status Scale.

a n (%); Mean (SD).

### Overall safety and tolerability

#### Glucose monitoring

The first 15 people in this study (5 in each arm) were monitored for the potential risk of hypoglycemia by testing a fingerstick blood glucose level at two time points within 90 ​min of the first dose of intranasal insulin. There were no bouts of hypoglycemia or differences between treatment groups (Table 3).

**Table 3:** Fingerstick blood glucose for first 15 participants.

Treatment arm	Intranasal insulin 20 ​international units (n ​= ​5)	Intranasal insulin 10 ​international units (n ​= ​5)	Placebo^a^ (n ​= ​5)
First timepoint, mean (SD)	97.8 (13.4)	95.8 (15.5)	90.0 (18.4)
Second timepoint, mean (SD)	88.4 (8.8)	92.2 (15.5)	87.8 (11.4)

Standard deviation (SD).

a One placebo participant was missing the second measurement.

#### Adverse events (AEs) and severe AEs

The most common relevant side effects (threshold for reporting ≥5 ​%) amongst the treatment groups included headache, rhinorrhea, and dizziness (Table 4). All study drug emergent AEs were transient in nature.

**Table 4:** Adverse events by systems per treatment arm (threshold for reporting ≥5 ​%).

Treatment arm	Placebo		Intranasal insulin 10 ​international units		Intranasal insulin 20 ​international units	
	Affected/at Risk (%)	# Events	Affected/at Risk (%)	# Events	Affected/at Risk (%)	# Events
**Total**	18/35 (51.43 ​%)	**18**	18/33 (54.55 ​%)	**18**	24/37 (64.86 ​%)	**24**
**Gastrointestinal disorders**						
Diarrhea	0/35 (0 ​%)	**0**	0/33 (0 ​%)	**0**	2/37 (5.41 ​%)	**2**
Dry mouth	0/35 (0 ​%)	**0**	1/33 (3.03 ​%)	**1**	2/37 (5.41 ​%)	**2**
**General disorders**						
Fatigue	1/35 (2.86 ​%)	**1**	2/33 (6.06 ​%)	**2**	1/37 (2.70 ​%)	**1**
Pain	4/35 (11.43 ​%)	**4**	1/33 (3.03 ​%)	**1**	2/37 (5.41 ​%)	**2**
**Infections & infestations**						
Upper respiratory infection	0/35 (0 ​%)	**0**	3/33 (9.09 ​%)	**3**	1/37 (2.70 ​%)	**1**
**Injury**						
Fall	1/35 (2.86 ​%)	**1**	1/33 (3.03 ​%)	**1**	2/37 (5.41 ​%)	**2**
Fracture	0/35 (0 ​%)	**0**	1/33 (3.03 ​%)	**1**	3/37 (8.11 ​%)	**3**
**Musculoskeletal**						
Arthralgia	2/35 (5.71 ​%)	**2**	0/33 (0 ​%)	**0**	1/37 (2.70 ​%)	**1**
Weakness	0/35 (0 ​%)	**0**	2/33 (6.06 ​%)	**2**	1/37 (2.70 ​%)	**1**
**Nervous system disorders**						
Dizziness	2/35 (5.71 ​%)	**2**	3/33 (9.09 ​%)	**3**	4/37 (10.81 ​%)	**4**
Headache	5/35 (14.29 ​%)	**5**	5/33 (14.29 ​%)	**5**	8/37 (21.62 ​%)	**8**
Memory impairment	1/35 (2.86 ​%)	**1**	0/33 (0 ​%)	**0**	2/37 (5.41 ​%)	**2**
Paresthesia	1/35 (2.86 ​%)	**1**	2/33 (6.06 ​%)	**2**	2/37 (5.41 ​%)	**2**
Presyncope	1/35 (2.86 ​%)	**1**	0/33 (0 ​%)	**0**	2/37 (5.41 ​%)	**2**
**Psychiatric disorders**						
Irritability	0/35 (0 ​%)	**0**	2/33 (6.06 ​%)	**2**	1/37 (2.70 ​%)	**1**
Nightmare	2/35 (5.71 ​%)	**2**	0/33 (0 ​%)	**0**	0/37 (0 ​%)	**0**
**Respiratory disorders**						
Nasal congestion	3/35 (8.57 ​%)	**3**	0/33 (0 ​%)	**0**	0/37 (0 ​%)	**0**
Rhinorrhea	3/35 (8.57 ​%)	**3**	4/33 (12.12 ​%)	**4**	3/37 (8.11 ​%)	**3**
Sore throat	1/35 (2.86 ​%)	**1**	0/33 (0 ​%)	**0**	4/37 (10.81 ​%)	**4**

There were 13 severe AEs distributed fairly equally amongst the treatment groups which were not deemed study drug related, and there were no deaths (Table 5).

**Table 5:** Serious adverse events per treatment arm.

Treatment arm	Placebo		Intranasal insulin 10 ​international units		Intranasal insulin 20 ​international units	
	Affected/at Risk (%)	# Events	Affected/at Risk (%)	# Events	Affected/at Risk (%)	# Events
**Total**	5/35 (14.29 ​%)	**5**	3/33 (9.09 ​%)	**3**	5/37 (13.51 ​%)	**5**
**Cardiac**						
Chest pain	0/35 (0 ​%)	**0**	0/33 (0 ​%)	**0**	1/37 (2.70 ​%)	**1**
**Gastrointestinal disorders**						
Small bowel obstruction	0/35 (0 ​%)	**0**	0/33 (0 ​%)	**0**	1/37 (2.70 ​%)	**1**
**Infections & infestations**						
Perirectal abscess	1/35 (2.86 ​%)	**1**	0/33 (0 ​%)	**0**	0/37 (0 ​%)	**0**
Urinary tract infection	1/35 (2.86 ​%)	**1**	1/33 (3.03 ​%)	**1**	0/37 (0 ​%)	**0**
Zoster ophthalmicus	1/35 (2.86 ​%)	**1**	0/33 (0 ​%)	**0**	0/37 (0 ​%)	**0**
**Injury**						
Fall	1/35 (2.86 ​%)	**1**	1/33 (3.03 ​%)	**1**	0/37 (0 ​%)	**0**
**Nervous system disorders**						
Dizziness	0/35 (0 ​%)	**0**	0/33 (0 ​%)	**0**	1/37 (2.70 ​%)	**1**
MS relapse	1/35 (2.86 ​%)	**1**	0/33 (0 ​%)	**0**	0/37 (0 ​%)	**0**
Worsening of neurological symptoms	0/35 (0 ​%)	**0**	0/33 (0 ​%)	**0**	2/37 (5.41 ​%)	**2**
**Psychiatric disorders**						
Non-epileptic seizures	0/35 (0 ​%)	**0**	1/33 (3.03 ​%)	**1**	0/37 (0 ​%)	**0**

Twenty-two subjects continued the study follow-up visits while off-medication; 9 discontinued treatment due to a variety of reasons, and 13 discontinued treatment due to the COVID-19 pandemic as instructed by the DSMB (see Methods section). In total, 12 subjects dropped out of the study early; 9 cited personal reasons, 1 had gastrointestinal symptoms, 1 had nightmares, and 1 had upper respiratory symptoms (sore throat and nose bleeds).

### Impact on outcome measures

**Fig. 3:**
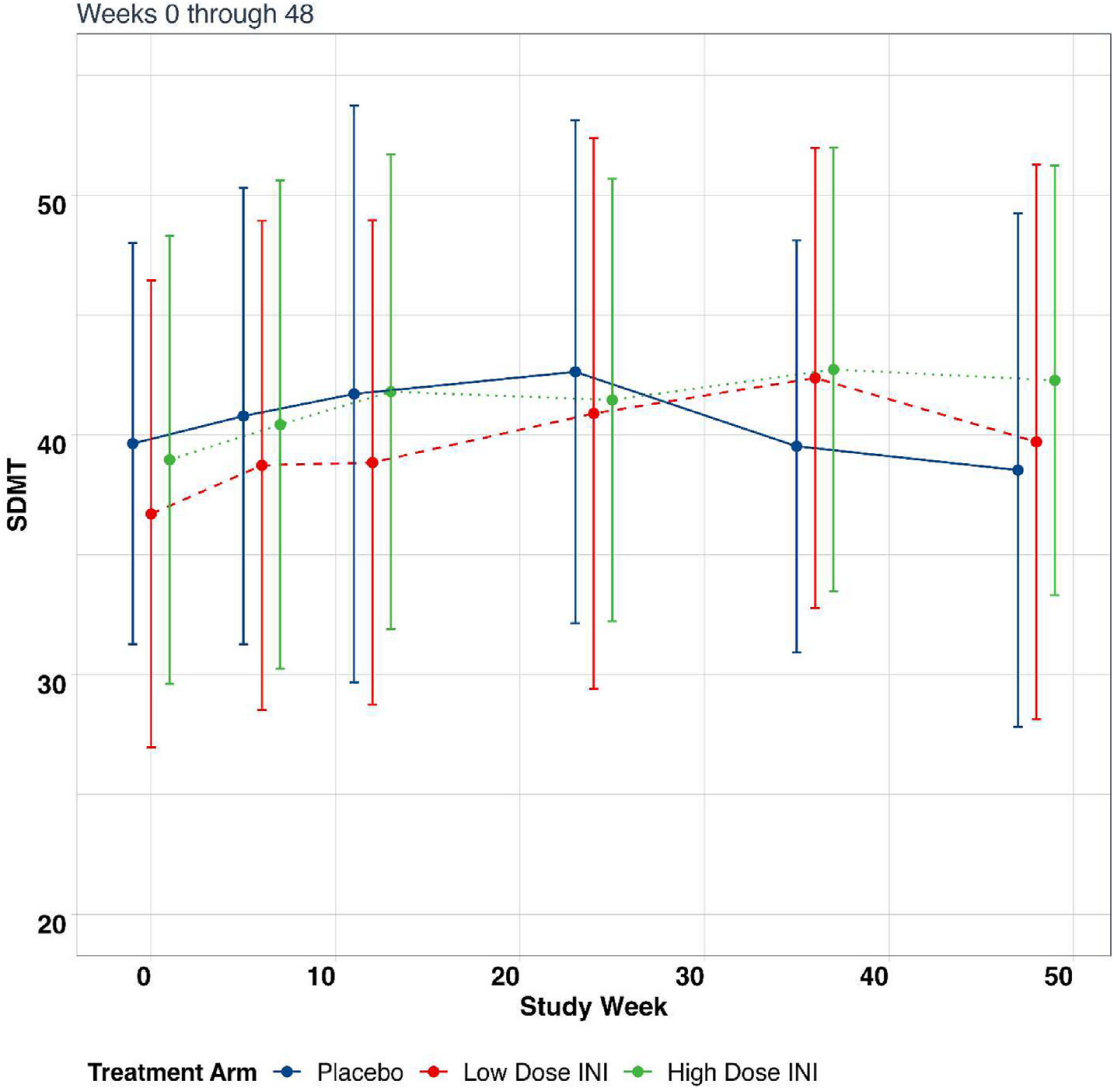
Mean change in SDMT per treatment arm throughout trial.

The primary clinical outcome measure, SDMT, did not demonstrate any difference between those treated with intranasal insulin (low or high dose) and placebo, (0.044 [−0.176, 0.263] p ​= ​0.70; −0.018 [−0.215, 0.180], p ​= ​0.86, respectively) (Table 6 and Fig. 3). This was the case even when stratifying by subtype of MS (relapsing or progressive MS).

**Table 6:** Difference in change in SDMT during 24-week intervention.

Treatment arm	N	Change/week (95 ​% CI)	P value	Difference in change/week vs. Placebo (95 ​% CI)	P value for difference
Placebo	32	0.163 (0.029, 0.297)	0.02		
Intranasal insulin 10 ​international units	30	0.207 (0.033, 0.381)	0.02	0.044 (−0.176, 0.263)	0.70
Intranasal insulin 20 ​international units	32	0.145 (0.000, 0.290)	0.05	−0.018 (−0.215, 0.180)	0.86

SDMT= Symbol Digit Modalities Test.

Similarly, the other components of MACFIMS did not demonstrate significant differences between active treatment and placebo (Supplemental Table).

The composite MSFC, individual components of MSFC, and EDSS remained stable over the course of the study for each treatment group.

Patient-reported outcomes (FAMS, FAMS subscales and PSQI) were not different between the treatment groups during the active treatment time.

### Per protocol specified analyses

Results were consistent in analyses in which we stratified by whether the person was enrolled during the COVID-19 or before, RRMS, disability status, age, disease modifying therapy use, depression history, and number of non-MS medications.

## Discussion

This phase Ib/II clinical trial is the first trial in MS to determine the safety and tolerability as well as the effectiveness of intranasal insulin as a therapeutic intervention for cognitive dysfunction. We demonstrated that intranasal insulin is safe and well-tolerated in PwMS. The most common side effects were transient in nature: headache, rhinorrhea, and dizziness. Similar findings were observed in a clinical trial assessing patients with mild cognitive impairment and AD [24]. The SAEs in our study were deemed unrelated to study drug and were distributed equally across the study treatment groups. Moreover, no deaths or study related treatment-emergent hospitalizations were seen.

Despite participants’ tolerating intranasal insulin, neither dose of intranasal insulin tested was superior to placebo in any of the clinical outcome measures assessed. It may be that this therapy is not effective for cognition in PwMS or there may be alternative explanations for the failure to reject the null hypothesis. The outcome measures used may not have been sensitive enough to detect subtle changes in cognitive function over time in PwMS. In fact, a recent study raised concerns about the ability of SDMT to detect cognitive change in a clinical trial, in part due to a strong practice effect [57]; indeed, there was some improvement during the study in all participant groups. On the other hand though, there was no apparent effect of intranasal insulin herein on other secondary measures of cognition, either. Our study may not have been long or large enough to detect subtle changes and overcome such practice effects, and data missingness, particularly given COVID-related impacts, may have led to reduced power or bias in the results. Finally, it is also possible that the dose chosen was insufficient, or that CNS penetration was inadequate. That being said, a recent phase 2 trial of intranasal insulin for AD began the trial using the device we used here but switched because of device malfunctions necessitating frequent replacements, and the intent to treat population was changed to focus only on those using the second device [58]. Surprisingly, while there was no apparent effect of intranasal insulin in those using the second device, in the small subcohort using the first device, there was an effect of intranasal insulin on some cognitive outcomes. While the disparate results may be spurious given the small size of the cohort, they also highlight the need for future studies to consider carefully mode of delivery. Furthermore, in people with cognitive impairment re-assessments of proper device use should be considered. In our study, participants were instructed to record the dates and times of each administered dose throughout the study and document if missed doses occurred. The study team kept in frequent contact with participants regarding their diary entries and met with those participants and caregivers (when relevant) who needed retraining on dose administration. Adherence proved to be high in our study based on diary entries and few participants required retraining, although it is possible participants had improper study drug administration technique outside of their witnessed administration by our study team and/or their adherence was not recorded accurately. Further, whether specific formulations of intranasal insulin have differing CNS penetration or clinical effects should be considered [59,60].

In trials of people with other cognitive issues, including mild cognitive impairment or AD, the effects of intranasal insulin on various outcome measures have been mixed. A recent meta-analysis of trials that included people with AD or mild cognitive impairment showed a benefit of 20 IU/day, with a trend for 40 IU/day, of intranasal insulin with respect to one outcome measure, the Alzheimer's Disease Assessment Scale-cognitive subscale (ADAS-cog), but not on several other cognitive measures [61]. Beyond the mild cognitive impairment/AD spectrum, intranasal insulin has shown benefits for cognition as well. For example, compared to placebo, perioperative intranasal insulin demonstrated a benefit with respect to post-operative cognitive performance and postoperative delirium in an older adult population undergoing elective surgery [62]. In a randomized controlled trial of more than 200 older adults with and without diabetes, intranasal insulin therapy was overall associated with improvements in cognition and gait compared to placebo, although differences comparing insulin-treated and placebo effects within diabetes or non-diabetes subgroups showed that the effects on cognitive domains were stronger in non-diabetics, and walking parameters seemed imbalanced among diabetics at baseline [63]. That results vary across studies likely reflects the heterogeneity of underlying conditions, outcome measures used, and participants included in the studies. That intranasal insulin continues to demonstrate potential benefits in some populations but did not show utility in MS may be related to some of the study design aspects above or due to differences in etiopathogenesis of cognitive problems in MS. Careful review of these more recently-published trials in other health conditions, and characterization of putative contributors to disparate results, would be useful prior to embarking on any future trials of intranasal insulin in people with MS in order to maximize the capacity to detect a treatment effect, if one exists.

There are several limitations of the current study that are worth noting, especially since they could have impacted the trial results. As previously mentioned, the study duration may have been too short, the intranasal insulin doses chosen inappropriate, and/or the outcome measures assessed were not sensitive enough to detect changes. Also, the sample size was small, especially with a three-arm trial design, and missing data during the active treatment phase of the trial could have led to reduced power to detect a treatment effect. Our participants may have been too cognitively impaired to see meaningful changes; perhaps intervening in people with fewer cognitive deficits and with a shorter history of cognitive complaints would be ideal. Further, our cohort had a relatively high level of education and thus potentially greater neurological reserve capacity, which could obscure a treatment effect. Additionally, pre-existing symptomatic therapies and mood disorders could have impacted the results, despite our attempt to control for such influences with our inclusion/exclusion criteria. At the same time, when enrollment was hindered by excluding people using such medications altogether, we also recognized that such exclusions would make the trial results much less generalizable, and that in all likelihood, therapies to improve cognition in people with MS would likely need to do so in spite of concomitant use of medications that may influence cognitive performance. Participants’ adherence to the study drug or administration instructions may have impacted the amount of insulin reaching the CNS from the intranasal device and thus have influenced the ability to detect an effect. Lastly, we did not have an imaging or specific fluid biomarker which might have demonstrated metabolic changes over time that eventually might impact cognitive function.

Despite the findings from our study, intranasally-administered therapeutics may be of interest to develop further as a way to get across the blood-brain barrier (BBB) [64]. An intranasal delivery system provides a non-invasive way to bypass the BBB and allow rapid delivery of a medication to the CNS via the olfactory and trigeminal perivascular channels [24,59,64]. The main advantage of this delivery system is reducing systemic side effects by limiting a medication's exposure to peripheral organs and tissues. In addition, first-pass metabolism is avoided. Further studies are needed to identify medications that can be delivered intranasally successfully and have a CNS biological effect that impacts clinical outcomes in MS.

## Author contributions

Scott D. Newsome: contributed to the conceptualization, methodology, investigation, research, writing first draft, reviewing and editing of the manuscript.

Kathryn C. Fitzgerald: contributed to the methodology, research, formal analysis, reviewing and editing of the manuscript.

Abbey Hughes: contributed to the investigation, research, reviewing and editing of the manuscript.

Meghan Beier: contributed to the investigation, research, reviewing and editing of the manuscript.

Jacqueline Koshorek: contributed to the investigation, research, reviewing and editing of the manuscript.

Yujie Wang: contributed to the investigation, research, reviewing and editing of the manuscript.

Daniela Pimentel Maldonado: contributed to the investigation, research, reviewing and editing of the manuscript.

Thomas Shoemaker: contributed to the investigation, research, reviewing and editing of the manuscript.

Taimur Malik: contributed to the investigation, research, reviewing and editing of the manuscript.

Tarik Bayu: contributed to the investigation, research, reviewing and editing of the manuscript.

Pablo E. Ravenna: contributed to the investigation, research, reviewing and editing of the manuscript.

Ama Avornu: contributed to the investigation, research, reviewing and editing of the manuscript.

Elias S. Sotirchos: contributed to the investigation, research, reviewing and editing of the manuscript.

Meghan Romba: contributed to the investigation, research, reviewing and editing of the manuscript.

John Muschelli: contributed to the investigation, research, reviewing and editing of the manuscript.

Todd T. Brown: contributed to the investigation, research, reviewing and editing of the manuscript.

Peter A. Calabresi: contributed to the conceptualization, research, reviewing and editing of the manuscript.

Ellen M. Mowry: contributed to the conceptualization, methodology, investigation, research, reviewing and editing of the manuscript.

## Declaration of competing interest

None.
