# Supplementary material for: Intranasal insulin for improving cognitive function in multiple sclerosis
Source: Neurotherapeutics. 2025 Apr 18;22(4):e00581. doi: 10.1016/j.neurot.2025.e00581 (PMC12418434; doi:10.1016/j.neurot.2025.e00581)
Supplement: Multimedia component 1 [file mmc1.docx]

**Supplemental Table:** Treatment impact on outcome measures in intention-to-treat groups

| Outcomes measures* | Within-group change over 24 weeks vs. baseline | | | Change over 24 weeks vs. baseline compared to placebo | |
| --- | --- | --- | --- | --- | --- |
|  | Placebo | Insulin 10 units BID | Insulin 20 units BID | Insulin 10 units BID | Insulin 20 units BID |
| Symbol Digit Modalities Test,  change/week (95% CI) [n] | 0.163  (0.029, 0.297) [n=32] | 0.207  (0.033, 0.381) [n=30] | 0.145  (0.000, 0.290) [n=32] | 0.044  (-0.176, 0.263) [n=30] | -0.018  (-0.215, 0.180) [n=32] |
| Paced Auditory Serial Addition Test-3,  change/week (95% CI) [n] | 0.212  (0.027, 0.398) [n=30] | 0.363  (0.136, 0.591) [n=29] | 0.372  (0.180, 0.563) [n=36] | 0.151  (-0.142, 0.444) [n=29] | 0.159  (-0.107, 0.426) [n=36] |
| Controlled Oral Word Association Test (COWAT),  change/week (95% CI) [n] | 0.021  (-0.174, 0.216) [n=35] | 0.070  (-0.162, 0.303) [n=31] | 0.090  (-0.112, 0.292) [n=37] | 0.049  (-0.243, 0.341) [n=31] | 0.069  (-0.198, 0.335) [n=37] |
| California Verbal Learning Test- II, change/week (95% CI) [n] | 0.020  (-0.043, 0.082) [n=35] | 0.021  (-0.057, 0.100) [n=31] | 0.082  (0.015, 0.149) [n=36] | 0.002 (-0.099, 0.102) [n=31] | 0.062 (-0.029, 0.154) [n=36] |
| Brief Visuospatial Memory Test, change/week (95% CI) [n] | 0.030  (-0.015, 0.075) [n=34] | 0.059  (0.006, 0.113) [n=29] | 0.027  (-0.020, 0.074) [n=36] | 0.029  (-0.041, 0.099) [n=29] | -0.003  (-0.068, 0.062) [n=36] |
| Judgement of Line Orientation,  change/week (95% CI) [n] | -0.005  (-0.079, 0.069) [n=35] | 0.047  (-0.044, 0.139) [n=29] | -0.031  (-0.109, 0.046) [n=36] | 0.052  (-0.066, 0.170) [n=29] | -0.026  (-0.133, 0.081) [n=36] |
| Delis-Kaplan Executive Function System sorting test,  change/week (95% CI) [n] | 0.002  (-0.114, 0.118) [n=32] | 0.027  (-0.104, 0.159) [n=29] | -0.001  (-0.112, 0.111) [n=35] | 0.025  (-0.150, 0.200) [n=29] | -0.003  (-0.164, 0.158) [n=35] |
| Beck Depression Inventory-II score, change/week (95% CI) [n] | -0.045  (-0.183, 0.094) [n=27] | -0.019  (-0.179, 0.140) [n=25] | -0.022  (-0.152, 0.108) [n=31] | 0.025  (-0.186, 0.237) [n=25] | 0.022  (-0.167, 0.212) [n=31] |
| Pittsburgh Sleep Quality Index Global Score,  change/week (95% CI) [n] | -0.045  (-0.110, 0.021) [n=33] | 0.035  (-0.035, 0.105) [n=30] | -0.026  (-0.091, 0.039) [n=34] | 0.080  (-0.016, 0.176) [n=30] | 0.019  (-0.074, 0.111) [n=34] |
| FAMS Global Score,  change/week (95% CI) [n] | 0.240  (-0.087, 0.567) [n=34] | 0.051  (-0.357, 0.459) [n=30] | 0.056  (-0.277, 0.389) [n=36] | -0.189  (-0.712, 0.334) [n=30] | -0.184  (-0.651, 0.283) [n=36] |

*Outcomes adjusted for age, sex and education status

**Supplemental Material:** Revised criteria and enrollment due to COVID-19 safety measures

Eligibility Criteria

1. Willing to comply with state/local recommended social distancing and other suggested COVID-19 related safety measures

Recruitment

1.For MS Center patients that have not taken an SDMT or PST test, we will use an oral consent process to administer the SDMT test and determine eligibility.

2. SDMT or PST test results obtained within 3 months of the baseline visit will be considered as proof of eligibility for the study and may be screened in person or by phone.

3. Modification of study visits/tasks

4. Removed CSF sub-study from the protocol due to a lack of interest in the substudy.

5. Weeks 12, 24, and 48 will no longer have both a physical exam and neurological exam, rather an EDSS only.

6. Due to COVID-19, many of the study tasks/visits will now be conducted remotely: a) Study consent will be reviewed at length virtually the day prior to, or the day of, the visit, with all questions asked at that time. b) Eligibility verification c) Medical hx, relapse assessment d) Medication review e) Adverse event assessment f) Pregnancy assessment g) Sleep questionnaires h) Neuro-Qol/Depression questionnaires i) Suicidality evaluation

7. Study visits at week 6, 36, and 48 are now conducted remotely.

8. No physical exams will be conducted.

9. If a severe COVID-19 outbreak occurs again such as occurred in the spring of 2020, we will conduct all follow-up assessments by video or phone until such time as it is safe to pursue the modified schedule, as above, or resume the originally-planned schedule.

10. Removed section on audio recording of MACFIMS

11. Exploratory Biomarkers - Plasma and/or PBMCs will be collected at baseline, week 12, and week 24 visits (except if COVID-19 related shutdown does not permit follow-up blood collection or lab-based processing).

12. We allowed more flexibility with respect to the amount of time to remotely consent subjects. Rather than virtually consenting at least a day prior to the in-person visit, we were approved to virtually consent at least a week prior to the baseline visit. Subjects can either sign the consent form at the time of virtual consent, if capable, or at the time of the baseline visit.
